# Identification of a Novel Delta Opioid Receptor Agonist Chemotype with Potential Negative Allosteric Modulator Capabilities

**DOI:** 10.3390/molecules26237236

**Published:** 2021-11-29

**Authors:** Yazan J. Meqbil, Hongyu Su, Robert J. Cassell, Kendall L. Mores, Anna M. Gutridge, Benjamin R. Cummins, Lan Chen, Richard M. van Rijn

**Affiliations:** 1 Department of Medicinal Chemistry and Molecular Pharmacology, College of Pharmacy, Purdue University, West Lafayette, IN 47907, USA; ymeqbil@purdue.edu (Y.J.M.); serenata@umich.edu (H.S.); rcassell@purdue.edu (R.J.C.); kendall.mores@nm.org (K.L.M.); Agutridg@purdue.edu (A.M.G.); 2 Computational Interdisciplinary Life Sciences, Purdue University, West Lafayette, IN 47907, USA; 3 Department of Chemistry, College of Science, Purdue University, West Lafayette, IN 47907, USA; bcummins96@gmail.com; 4 Purdue Institute for Drug Discovery, Purdue University, West Lafayette, IN 47907, USA; chen2178@purdue.edu; 5 Purdue Institute for Integrative Neuroscience, Purdue University, West Lafayette, IN 47907, USA

**Keywords:** chemotype, high-throughput screen, delta opioid receptor, allosteric modulation, beta-arrestin, molecular dynamics

## Abstract

The δ-opioid receptor (δOR) holds great potential as a therapeutic target. Yet, clinical drug development, which has focused on δOR agonists that mimic the potent and selective tool compound SNC80 have largely failed. It has increasingly become apparent that the SNC80 scaffold carries with it potent and efficacious β-arrestin recruitment. Here, we screened a relatively small (5120 molecules) physical drug library to identify δOR agonists that underrecruit β-arrestin, as it has been suggested that compounds that efficaciously recruit β-arrestin are proconvulsant. The screen identified a hit compound and further characterization using cellular binding and signaling assays revealed that this molecule (R995045, compound **1**) exhibited ten-fold selectivity over µ- and κ-opioid receptors. Compound **1** represents a novel chemotype at the δOR. A subsequent characterization of fourteen analogs of compound **1**, however did not identify a more potent δOR agonist. Computational modeling and in vitro characterization of compound **1** in the presence of the endogenous agonist leu-enkephalin suggest compound **1** may also bind allosterically and negatively modulate the potency of Leu-enkephalin to inhibit cAMP, acting as a ‘NAM-agonist’ in this assay. The potential physiological utility of such a class of compounds will need to be assessed in future in vivo assays.

## 1. Introduction

**Figure 1:**
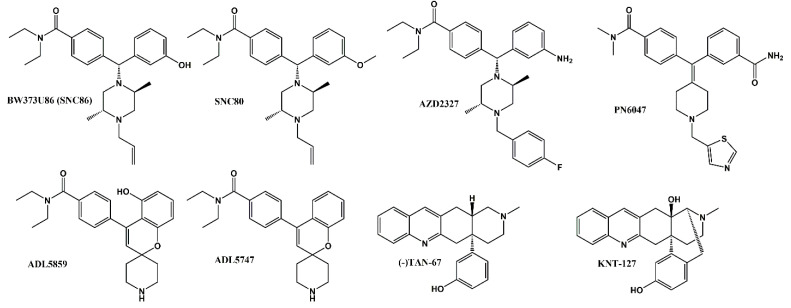
Chemical structure of δOR agonists. BW373U86 (SNC86), SNC80, AZD2327, PN6047, ADL5859, ADL5747, (-)TAN-67 and KNT-127.

The δ-opioid receptor (δOR) has great potential as a therapeutic target to treat a myriad diseases and disorders. Preclinical use of δOR agonists suggest their utility to reduce anxiety, depression, alcohol use, migraine, neuropathic and inflammatory pain [1,2]. Yet, to this day roughly 30 years since the δOR was cloned [3,4] no δOR selective molecule has been FDA approved for clinical use. Between 2008–2010 a small set of δOR agonists entered phase II clinical trials (NCT00993863, NCT01058642, NCT00759395 and NCT00979953) for acute and chronic pain conditions as well as to treat depressive disorders [5]. However, none of these trials progressed to phase 3 clinical trials. A common shared feature of the phase 2 drug candidates, ADL5859, ADL5747 and AZD2327 was that their structure was based on that of previously developed potent and highly selective δOR agonists SNC80 and BW373U86 (SNC86), (Figure 1, [6,7,8,9,10]). A major concern with the original SNC compounds was their propensity to induce severe seizures in rodents [11]. AZD2327 exhibited proconvulsant effects [8], whereas Adolor was able to modify the SNC structure enough to not observe tonic-clonic seizures [6,7]. However, recent studies have led to a better understanding of the mechanism by which SNC80 can cause seizures, implicating β-arrestin as a critical factor [12,13]. The SNC compounds are super-recruiters of β-arrestin, and it appears that ADL5859 and AZD2327, recruit β-arrestin on par with the endogenous agonist Leu-enkephalin, if not stronger [14,15,16]. In 2020, Conibear et al., developed a novel δOR agonist PN6047 (Figure 1), based on the SNC80 scaffold, which was not proconvulsant, and which recruited β-arrestin with efficacy slightly less than ARM390 (which in our hands has an Emax on par with Leu-enkephalin) [14,17]. Thus, PN6047 shared similarity with the failed Adolor and Astra Zeneca compounds, looking promising in terms of preclinical in vivo effects, but retaining high risk for a failure once moved into human clinical trials. Thus, in order for the δOR field to progess and produce a clinically viable candidate it is important to divert from the SNC80 scaffold. A handful of δOR selective small molecules have been produced that suggest this is possible: TAN-67 and KNT-127 (Figure 1) have distinct scaffolds and under-recruit β-arrestin, respectively with Emax for β-arrestin 2 recruitment of 30%, 70% and do not induce convulsions [14,18,19]. Similarly, kratom alkaloids, while displaying pan-opioid activity, are highly G-protein biased in that they do not show detectable β-arrestin 2 recruitment [20]. Our goal for this study was to identify novel δOR agonist scaffold(s) that under-recruit β-arrestin (relative to SNC80). In this study, we screened over 5000 chemical compounds from CNS-focused drug libraries. We were able to identify a molecule (compound **1**) with a novel chemical scaffold that was selective for δOR over the µ- and κ-opioid receptors (µOR and κOR) with micromolar affinity and potency. Computational modelling of compound **1** into the δOR crystal structure (PDB: 6PT3) suggests it is able to partially occupy the known orthosteric binding pocket as well as an allosteric binding pocket in the presence of Leu-enkephalin. Further in vitro analysis showed that compound **1** potentially negatively modulates the potency of Leu-enkephalin in an allosteric manner.

## 2. Results

### 2.1. Identification of a Novel δOR Agonist with Sub-Maximal β-Arrestin Recruitment Efficacy

**Figure 2:**
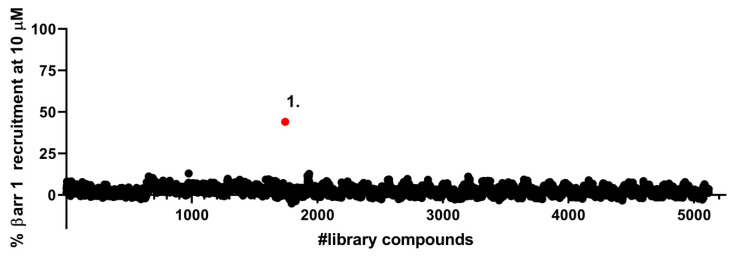
Screening of a CNS-targeted compound library for β-arrestin 1 recruitment at δOR. 5200 compounds from sixteen 384-well plates from diverse CNS-targeted drug libraries were tested at 10 µM for β-arrestin 1 recruitment at δORs in a PathHunter assay. The red dot represents the hit compound (**1**). 10 µM SNC80 was utilized for normalization.

We have previously reported that SNC80 super-recruits β-arrestin 2 relative to Leu-enkephalin but has equal β-arrestin 1 recruitment efficacy [14,15]. Thus, for ease of setting a cut-off threshold we decided to perform a high-throughput screen with the β-arrestin 1 cells. We tested ~5100 compounds and identified a single positive hit, that, at a 10 µM concentration, displayed ~50% β-arrestin 1 recruitment relative to SNC80 (Figure 2).

### 2.2. Compound ***1*** Displays 10-Fold Selectivity over µOR and κOR

**Figure 3:**
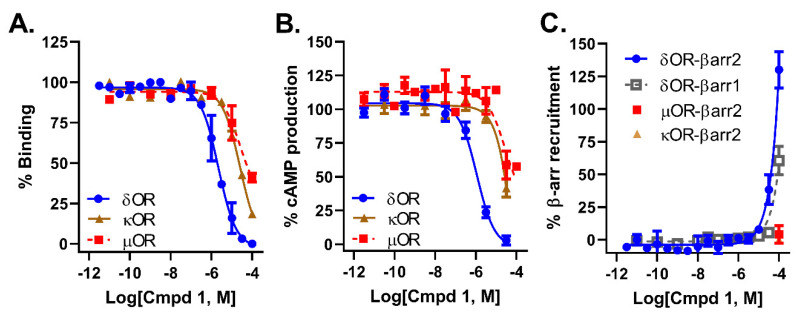
Pharmacological characterization of compound **1**. (**A**). Binding affinity for compound **1**. At δOR, µOR and κOR. (**B**). Inhibition of forskolin induced cAMP by compound **1** in cells expressing δOR, µOR and κOR. (**C**). β-arrestin recruitment for compound **1** following stimulation of δOR, µOR and κOR.

Pharmacological characterization of compound **1** revealed that it had a micromolar affinity (Figure 3A) and potency (Figure 3B) at the δOR, which was roughly 10-fold stronger than for the µOR and κOR (Table 1). Within the testable dose range (<100 µM) we were unable to detect any β-arrestin 2 recruitment for compound **1** at the µOR and κOR (Table 1, Figure 3C). At the highest concentration we were able to detect β-arrestin 1 and 2 recruitment at the δOR (Figure 3C), but we were unable to generate pEC_50_ or alpha values in these assays as we had not reached the maximum effect yet.

### 2.3. Compound ***1*** Derivatives Exhibit Lower δOR Potency

**Figure 4:**
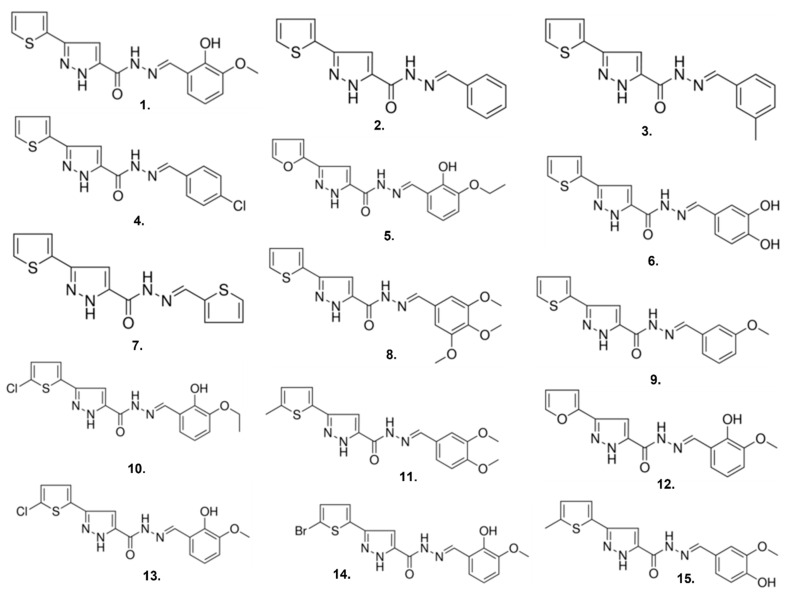
Chemical structures of compound **1** and **14** analogs of compound **1**.

The hit compound (**1**), *N*’-(2-hydroxy-3-methoxybenzylidene)-3-(2-thienyl)-1*H*-pyrazole-5-carbohydrazide, had a novel chemotype and in contrast to well-established δOR agonists Leu^5^-enkephalin, SNC80 and ADL5859 appears to lack a basic nitrogen. Next, we performed a structure activity relationship (SAR) by catalog using 14 analogs of compound **1** (Figure 4, Table 2) to investigate how compound **1** may bind to δOR and to possibly identify compounds with improved pharmacology. In our experience, potency for δOR agonism in the PathHunter β-arrestin assay is generally lower than for the cAMP assay [21]. Therefore, to assess if analogs of compound **1** displayed improved δOR potency we first characterized the compounds in the cAMP assay. We found that none of the purchased analogs had stronger potency for δOR activation than compound **1** (Table 2).

### 2.4. Compound ***1*** Engages Amino Acid Residues That Form the Orthosteric Binding Pocket

**Figure 5:**
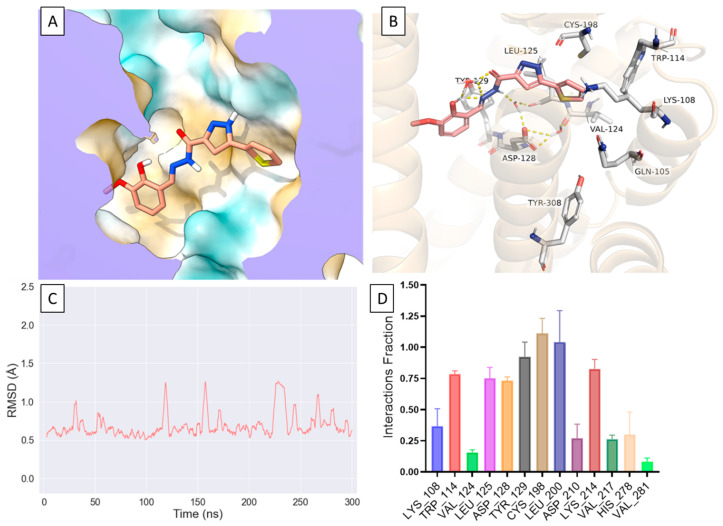
Molecular Dynamic simulation of Compound **1** binding to the δOR. (**A**). Compound **1** bound at δOR where its positioned within the hydrophobic pocket, a predicted allosteric site. (**B**). Compound **1** interacts with residues forming the hydrophobic pocket as well as with residues deeper into the orthosteric site K108^2.63^, W114^ECL1^, L125^3.29^, D1283^.32^, Y129^3.33^, C198^ECL2^, L200^ECL2^ and K214^5.40^. (**C**). A rolling average of 3 ns of the RMSD of compound **1** in a 300 ns MD simulation showing a relatively stable binding pose for compound **1**. (**D**). Interaction fractions between compound **1** and the δOR in 3 different MD simulations.

Given the novelty of compound **1**′s scaffold, we wanted to model possible interactions of compound **1** at the δOR. We utilized the active-like crystal structure of δOR (PDB: 6PT3 [22]) to perform docking and molecular dynamics (MD) simulations in Schrödinger 2021-1. The crystal structure (6PT3) contains nine thermostabilizing mutations, three of which are near at the sodium binding pocket (N 90^2.45^, D 95^2.50^, N 131^3.35^) and near ECL2 in transmembrane helix 2 (TM2) (Q105^2.60^ and K108^2.62^). Subsequently, we reverted all nine mutations to the wild-type (WT) residues (see methods and Supplementary Material). Our initial docking suggested that the thiophene ring of compound **1** occupies a hydrophobic pocket near the orthosteric site formed by W114^ECL1^, V124^3.28^, L125^3.29^, C198^ECL2^ where it forms ionic bonds with K108^2.63^ and hydrophobic interactions with W114^ECL1^ and C198^ECL2^ (Figure 5A). Additionally, compound **1** appeared to extend further into the orthosteric site where it was in proximity to and interacted with D128^3.32^, Y129^3.33^ and Y308^7.42^ (Figure 5B). To confirm the initial docked poses, we docked compound **1** into multiple potential binding sites generated using SiteMap and confirmed similar interactions with residues within the hydrophobic pocket (Supplementary Figure S1). We then decided to further model the interactions of compound **1** at δOR using dynamic structures where we performed three independent all-atom MD simulations which showed a relatively stable pose for compound **1** where it interacts with residues in TM2, ECL1, TM3 and ECL2 (L200^ECL2^) and occasionally with residues in TM5 (K214^5.40^) and TM7 (Figure 5C,D, Supplementary Figures S2 and S3).

### 2.5. Compound ***1*** Can Occupy an Allosteric Space alongside Leu-Enkephalin

**Figure 6:**
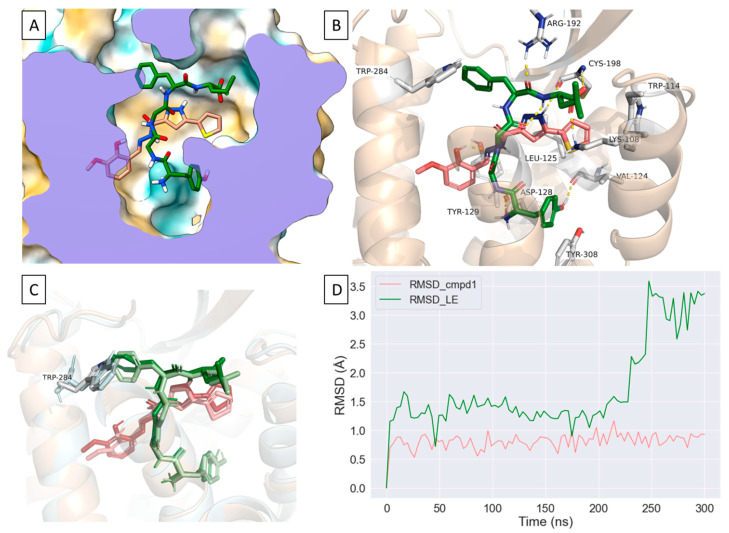
Molecular dynamic simulation of Compound **1** bound to the δOR in the presence of Leu-enkephalin. (**A**). a representative binding pose for compound **1** in the presence of Leu-enkephalin obtained from a 300 ns MD simulation where compound **1** stably occupies the partially hydrophobic pocket. (**B**). Leu-enkephalin forms H-bonds and water mediated interactions with K108^2.63^, D128^3.32^, R192^ECL2^, C198^ECL2^, H301^7.35^, C303^7.37^ and hydrophobic interactions with Y308^7.42^ whereas compound **1** mostly interacts with W114^ECL1^, L125^3.29^, C198^ECL2^ and L200^ECL2^ and K214^5.40^. (**C**). Poses of Leu-enkephalin and compound **1** showing the first frame of a 300 ns MD simulation (Leu-enkephalin: light green, compound **1**: light pink, W284: cyan) aligned on the clustered poses of Leu-enkephalin and compound **1** (Leu-enkephalin: dark green, compound **1**: red, W284^6.58^: light grey). (**D**). A rolling average of 3 ns of the RMSD of compound **1** in the presence of Leu-enkephalin obtained from a 300 ns MD simulation showing a relatively stable pose for compound **1** whereas the disruption of Leu-enkephalin’s interaction with W284^6.58^ causes a relatively large change in its RMSD.

Our modeling suggests that compound **1** interacts with residues in TM2 and TM7, which have been previously reported to interact, potentially, with the positive allosteric modulator BMS 986187 [23]. At the orthosteric site, compound **1** forms water-mediated interactions, hydrogen bonds and hydrophobic interactions with D128^3.32^, Y129^3.33^ and H278^6.62^ residues which were reported to be involved in δOR activation [22]. Additionally, compound **1** interacts with W114^ECL1^ (π-π stacking), V124^3.28^, L125^3.29^, C198^ECL2^ where its thiophene moiety occupies a partially hydrophobic pocket that is adjacent to the orthosteric site (Figure 5A). These unique interactions which include amino acid residues in the orthosteric and the potential allosteric binding sites prompted us to model compound **1** in the presence of Leu-enkephalin using molecular dynamics (MD) simulations (Figure 6A, Supplementary Figure S4). Intriguingly, compound **1** appears to maintain a relatively stable orientation as shown by the relatively stable RMSD in three independent MD simulations whereas Leu-enkephalin undergoes more dramatic confirmational changes in the presence of compound **1** (Figure 6B–D). Specifically, the presence of compound **1** appears to disrupt the π-π interaction between Leu-enkephalin with W284^6.58^ where the phenyl group of Phe^4^ rotates away from W284^6.58^ (Figure 6C). We also observed an inward shift in ICL2 as well as conformational changes at the intracellular side of δOR in ICL2, TM5 and TM6 when compared to the thermostabilized crystal structure (Supplementary Figure S5).

### 2.6. Compound ***1*** Potentially Negatively Modulates Potency of Leu-Enkephalin through an Allosteric Mechanism

**Figure 7:**
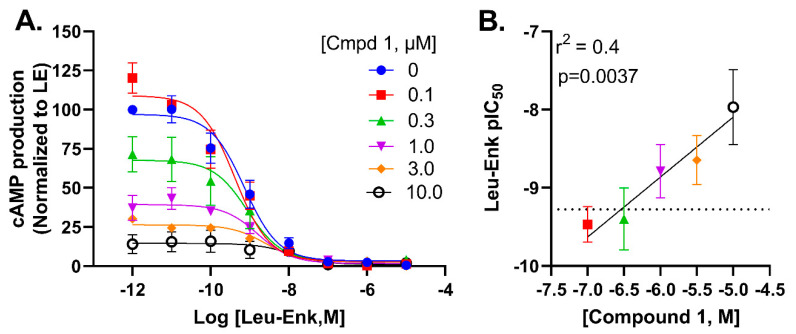
Compound **1** acts as a negative allosteric modulator for leu-enkephalin potency in the cAMP glosensor assay. (**A**). Dose-dependent inhibition of forskolin-mediated cAMP production by Leu-enkephalin (Leu-Enk) in the absence or presence of increasing concentrations of compound **1**. (**B**). The decrease in Leu-enkephalin pIC50 is correlated with increasing concentration of compound **1**.

Given that our modelling efforts suggested binding poses in a slightly allosteric binding pocket, we next decided to measure to what degree compound **1** modulated the activity profile of leu-enkephalin in the cAMP glosensor assay. We noted an increase in baseline (or τ_β_) when Leu-enkephalin was co-incubated with increasing concentrations of compound **1** (Figure 7A,B), without observing a chance in E_max_ (β = 1). We observed a left-shift in Leu-enkephalin potency suggestive of a negative allosteric modulation that is affinity (or α) driven (Figure 7A,B). As such, compound **1** appears to act as a negative allosteric modulator (NAM)-agonist [24] in the cAMP glosensor assay. It is well known that, for example, irreversible antagonists by lowering the receptor reserve will right-shift the potency of an agonist [25]. Thus, the potency shift could also be driven by the decrease in receptors available for Leu-enkephalin to bind to since radioligand binding indicates that compound **1** can bind and displace agonists (Figure 3) from the binding pocket.

## 3. Discussion

**Figure 8:**
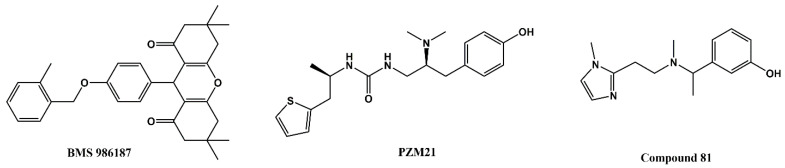
Chemical structures of the allosteric G-protein-biased δOR modulator BMS 986187, the G-protein-biased µOR agonist PZM21 and the G-protein-biased κOR agonist compound 81.

Here we report on a novel δOR-selective agonist chemotype that was identified from a 5120-compound high-throughput screen of CNS-targeted chemical libraries. The scaffold lacks a basic protonated amine, which is generally considered a hallmark feature of opioid ligands, needed to form a stable salt-bridge with aspartate D^3.32^ [22]. Using MolgpKa [26], the predicted pKa of the basic nitrogen in the pyrazole ring of compound **1** is 1.4, in sharp contrast with the pKa for protonated basic amines that is closer to physiological pH. A second interesting feature of compound **1** is the apparent negative allosteric modulation of the endogenous agonist Leu-enkephalin. Positive allosteric modulators (PAMs) have been identified for the opioid receptors, including the G-protein-biased δOR ‘PAM-agonist’ BMS 986187 (Figure 8) [24,27,28,29]. Cannabidiol and tetrahydrocannabinol have been proposed to be allosteric modulators of the δOR, specifically accelerating naltrindole dissociation rate [30], however to our knowledge no NAM-agonist has previously been reported.

The PAM-agonist BMS 986187 does not possess an ionizable group and thus resembles our compound **1**, which also lacks the protonated amine commonly present in opioids. However, comparisons between the suggested mode of binding of BMS 986187 and compound **1** at δOR show distinct interactions that could account for the differences in their mode of action. Notably, in the presence of the endogeneous peptide Leu-enkephalin, compound **1** appears to occupy a partially hydrophobic pocket adjacent to the orthosteric site which allows compound **1** to interact with residues in ECL1 (W114^ECL1^), ECL2 (C198, L200) and TM7, whereas BMS 986187 is reported to interact with residues in TM2 and TM7 in its lowest relative free-energy state in the presence of SNC80 [23]. Moreover, most of the residues reported to interact with BMS 986187 were shown to interact with residues in the active-like structures of δOR that constitute the orthosteric binding site [22,23]. These differences in the interactions could account for the distinct pharmacology of compound **1** and BMS 986187. Intriguingly, in the presence or absence of Leu-enkephalin, compound **1** maintains a relatively stable orientation that enables it to retain its hydrophobic and water-mediated interactions at the thiophene and pyrazole rings, respectively (Figure 6A,D). The presence of Leu-enkephalin, however, appears to disrupt the water-mediated interactions between compound **1** and orthosteric residues D128^3.32^ and Y129^3.33^ (Figure 6B) and changes the number of hydrogen donors or acceptors in compound **1** (Supplementary Figure S5). On the other hand, the presence of compound **1** disrupts the hydrophobic interaction between Phe^4^ and δOR by causing the phenyl group of Leu-enkephalin to rotate away from the side chain of W284^6.58^ (Figure 6C). Additionally, H-bond and water-mediated interactions between Leu-enkephalin and R192^ECL2^ appear to move ECL2 toward Leu-enkephalin which could open a cryptic binding site similar to a previously reported allosteric binding site in the angiotensin II (AngII) type 1 receptor [31] (Figure 6B, Supplementary Figure S6). As such, we predict that compound **1** may induce NAM activity by either destabilizing Leu-enkephalin or by playing an analogous role to BMS 986187 where it stabilizes the Na^+^ binding at δOR which increases the likelihood of receptor deactivation. It should be noted that comparisons between the binding modes of compound **1** and BMS 986187 at the δOR are limited due to the differences in the crystal structures used for modeling (agonist-bound vs antagonist-bound, respectively), chemotype differences between compound **1** and BMS 986187, the modeling method utilized, and the co-simulated ligand. Hence, future studies should examine the binding of compound **1** at the δOR in the presence of small molecule agonists and the implementation of enhanced sampling methods to model its interactions in the presence or absence of δOR agonists.

After identifying compound **1** in our screen, we had hoped to find analogs with higher potency, through a SAR by catalog. However, none of the purchased analogs displayed improved potency for the δOR. Our choice of catalog analogs was driven primarily by price and availability and much less guided by intelligent design. As a result of this strategy, we were only able to explore minor derivatization at the thiopene moiety and the 2-hydroxy-3-methoxybenzene moiety. Therefore, it is possible that compound **1** may still be improved on, for example, by altering or substituting on the pyrazole group, or by adding hydrogen bond-forming and/or accepting groups on the thiophene moiety.

Another feature we set out to find in our screen was a δOR agonist that underrecruited β-arrestin. Much effort has been devoted to identify opioids that display a preference to recruit and activate G-proteins relative to β-arrestin recruitment [17,21,32,33,34]. Our screen was designed with the purpose of finding molecules that underrecruit β-arrestin, but that are not G-protein-selective i.e., that entirely avoid β-arrestin recruitment and as such, compound **1** does still recruit β-arrestin. Surprisingly, we noted an unusual steep increase in β-arrestin recruitment at the δOR when stimulated with 100 µM compound **1**, such that we were unable to accurately predict an Emax. The sharp rise in β-arrestin recruitment at 100 µM did not appear to be a pan- interference assay effect, as we did not observe a similar response in our µOR and κOR PathHunter cell lines (Figure 3C). The mechanism or implication of compound **1**′s β-arrestin recruitment at 100 µM will require further investigation.

With increased availability of apo-state, antagonist-bound and agonist bound opioid structures, drug screening has moved away from screening physical libraries to screening virtual libraries. A computational model created using the crystal structure of an antagonist bound κOR [35] supported a virtual chemical library screen of 5 million molecules at κOR resulting in the identification of compound 81 (Figure 8), which is a G-protein-biased agonist with an 0.16 µM affinity and 0.53 µM potency at the κOR [36]. A virtual screen of 3 million molecules docked at a computational model of the µOR based on the antagonist-bound µOR crystal structure [37] resulted in the identification of a hit with 2.5 µM affinity at the µOR, which through an analog screen was improved to a lead compound with a 42 nM affinity and G protein bias. Further structure guided optimization of the lead compound resulted in the design of PZM21 (Figure 8), a G-protein-biased µOR-selective agonist with 1 nM affinity and unique chemotype [32]. Recent advances now allow for virtual screening of libraries containing more than a billion compounds [38,39]. While it is undeniable that large virtual screens can identify completely novel chemical matter, the ability to discover molecules with novel pharmacology may be more limited or biased by the type of structure (e.g., an orthosteric agonist-bound structure stabilized by a heterotrimeric G-protein or nanobody-mimic in a single active conformation) used for docking. Thus, in conclusion, our results highlight a current persisting value of chemical library screens in identifying molecules with unique binding modes and pharmacology.

## 4. Materials and Methods

### 4.1. Chemicals

Leu^5^-enkephalin, compounds **1**–**15** and forskolin were purchased from Sigma-Aldrich (St. Louis, MO USA). [D-Ala^2^, N-MePhe^4^, Gly-ol] enkephalin (DAMGO), SNC80 and U50,488 were purchased from Tocris Bioscience (Minneapolis, MN, USA). Radiolabels were from Perkin Elmer (Waltham, MA, USA).

### 4.2. Library Screen

In consultation with the Chemical Genomics Facility within the Purdue Institute for Drug Discovery, we screened sixteen 384-well plates that were part of CNS-targeted drug libraries. Specifically, we screened eleven plates part of a CNS-Chemdiv library, three plates part of a Chembridge ion channel library, and two plates part of a CNS-TimTec library. Each plate contained 320 compounds and four spare columns that were utilized to run positive (10 µM SNC80, 32 wells) and negative controls (0.02% DMSO, 32 wells), which were used to calculate Z-factors (average: Z’= 0.53, hit plate: Z’= 0.58) and normalize the data across plates. Using an Echo 525 acoustic liquid handler (Labcyte, San Jose, CA, USA), depending on the stock concentration (1, 10 or 20 mM) of the library plate 5, 10 or 100 nL of each compound was transferred from the library plate to the assay plate, the final concentration of each library compound was 10 µM.

### 4.3. Radioligand Binding Assay

Radioligand binding was performed as previously described [40,41]. For the binding assay 50 µL of a dilution series of peptide was added to 50 µL of 3.3 nM [^3^H]DPDPE (*K*_d_ = 3.87 nM) or 2.35 nM of [^3^H]DAMGO (*K*_d_ = 1.07 nM) or 0.8 nM of [^3^H]U69,593 (*K*_d_ = 1.2 nM) in a clear 96 well plate. Next, 100 µL of membrane suspension containing 7 µg protein was added to the agonist wells and incubated for 90 min at room temperature. The reaction mixture was then filtered over a GF-B filter plate (Perkin Elmer) followed by four quick washes with ice-cold 50 mM Tris HCl. The plate was dried overnight, after which 50 µL scintillation fluid (Ultimagold uLLT) was added and radioactivity was counted on a Packard TopCount NXT scintillation counter. All working solutions were prepared in a radioligand assay buffer containing 50 mM Tris HCl, 10 mM MgCl_2_, and 1 mM ethylenediaminetetraacetic acid at pH 7.4.

### 4.4. Cellular Signaling Assays

cAMP inhibition and β-arrestin 1 and 2 recruitment assays were performed as previously described [18]. In brief, for cAMP inhibition assays HEK 293 (Life Technologies, Grand Island, NY, USA) cells were transiently transfected in a 1:3 ratio with FLAG-mouse δOR, or HA-mouse µOR and pGloSensor22F-cAMP plasmids (Promega, Madison, WI, USA) using Xtremegene9 (Sigma). Two days post-transfection cells (20,000 cells/well, 7.5 µL) were seeded in low volume Greiner 384-well plates (#82051-458, VWR, Batavia, IL, USA) and were incubated with Glosensor reagent (Promega, 7.5 µL, 2% final concentration) for 90 min at room temperature. Cells were stimulated with 5 µL drug solution for 20 min at room temperature prior to stimulation with 5 µL forskolin (final concentration 30 µM), for an additional 15 min at room temperature. For β-arrestin recruitment assays, CHO-human µOR PathHunter β-arrestin 2 cells, CHO-human δOR PathHunter β-arrestin 2 cells, U2OS κOR PathHunter β-arrestin 2 cells or U2OS PathHunter β-arrestin 1 cells (DiscoverX, Fremont, CA, USA) were plated (2500 cells/well, 10 µL) one day prior to stimulation with 2.5 µL or 5–100 nL (in the screen) drug solution for 90 min at 37 °C/5% CO_2,_ after which cells were incubated with 6 µL cell PathHunter assay buffer (DiscoverX) for 60 min at room temperature as per the manufacturer’s protocol. Luminescence for each of these assays was measured using a FlexStation3 plate reader (Molecular Devices, Sunnyvale, CA, USA). As positive control we utilized Leu^5^-enkephalin or SNC80 (in the screen) for δOR, [D-Ala^2^, N-MePhe^4^, Gly-ol] enkephalin (DAMGO) for µOR and U50,488 for κOR.

### 4.5. Assessment of Allosteric Modulation

We ran log-step concentration response curves for Leu-enkephalin (10 µM–1 pM) in the presence of 0, 0.1, 0.3, 1, 3, or10 µM compound **1** in the δOR glosensor cAMP assay.

### 4.6. Data and Statistical Analysis

All data are presented as means ± standard error of the mean, and analysis was performed using GraphPad Prism 8 software (GraphPad Software, La Jolla, CA, USA). For in vitro assays, nonlinear regression was conducted to determine pIC_50_ (cAMP) or pEC_50_ (β-arrestin recruitment). Technical replicates were used to ensure the reliability of single values, specifically each data point for binding and β-arrestin recruitment was run in duplicate, and for the cAMP assay in triplicate. The averages of each independent run were counted as a single experiment and combined to provide a composite curve in favor of providing a ‘representative’ curve.

### 4.7. Receptor and Ligand Preparation for Molecular Modeling

The crystal structure of the active-like δOR (PDB: 6PT3) bound to small molecule agonist, DPI-287, was obtained from the Protein Data Bank (PDB) [22]. Molecular modeling was performed via Maestro (Schrödinger suite 2021-1, Schrödinger, Inc., New York, NY, USA). The *Protein Preparation Wizard* was used to prepare the structures before docking. The crystal structure was preprocessed to cap the N-terminus, remove the BRIL tag, membrane lipids and other crystal waters or ions not involved in mediating receptor-ligand interaction. Preliminary modeling and energy minimization of the thermostabilized receptor [22] and the WT-reverted receptor (data not shown) showed the feasibility of performing MD simulations using a truncated version of the WT receptor (residues 41–289) where all 9-thermostabilizing mutations were reverted to the WT (Supplementary Figure S5). Missing loops and side chains in the crystal structure were modeled using *Prime* within Schrödinger [42,43,44]. H-bond were assigned using the PROPKA algorithm [45,46]. All-atom MD simulations were performed on the modeled receptor using Desmond (Schrödinger, Inc.) implementing the OPLS4 force field. Compound **1** was prepared using LigPrep where the ionization states were assigned using Epik at pH 7.0 ± 2.0 [47,48]. Docking grids were generated for a representative structure from the MD simulations using *Receptor Grid Generation* in Schrödinger Release 2021-1 (Schrödinger, Inc.) using default parameters.

### 4.8. Ligand Docking Using Glide

Compound **1** and a set of known δOR ligands (Supplemental Table S1) were docked into a model WT δOR using Glide (Supplemental Table S2) [49,50,51]. Further structural optimization was needed to improve the docking accuracy of the model WT δOR (Supplemental Table S3). Additionally, given the novelty of the compound **1**′s chemotype, δOR ligands were docked into several models with predicted binding sites that were generated using SiteMap [52,53]. The best model was selected for further production MD simulations. Standard precision (SP) scoring function in Schrodinger 2021-1 was used for the initial docking of the molecules. The extra precision (XP) scoring function was then to further refine the docked poses. Post-docking energy minimization was performed for the top 50 poses of each small molecule, after which top 10 poses were visually inspected. The top 50 docked poses were also scored using Prime MM-GBSA scoring [54]. The best pose (based on docking, visual inspection and MM-GBSA score) was selected for subsequent production MD simulations (Supplemental Tables S4–S6).

### 4.9. Molecular Dynamics Simulations of Compound ***1*** at δOR

Production molecular dynamics simulations (MD) were performed in Desmond as reported previously [55]. Ligand-receptor complexes were embedded in a POPC membrane contained in a SPC-solvated orthorhombic box while maintaining a 10 Å distance from box boundaries. Na^+^ and Cl^−^ ions at a concentration of 0.15 M were added to mimic biological conditions using System Builder in Schrodinger 2021-1. The default membrane relaxation protocol in Desmond was used for membrane relaxation. Then a constant pressure and temperature (NPT) equilibration run was performed for 100 ns. The RESPA integrator with a 2 fs integration step for bonded interactions and a 6 fs step for non-bonded interactions. The Nosé-Hoover thermostat (and Martyna-Tobias-Klein barostat with semi-isotropic coupling to maintain temperature at 300 K and pressure at 1 bar. For the production MD simulations, three independent 200 ns NPT simulations were carried out for compound **1** in complex with modeled δOR or compound **1** and Leu-enkephalin in complex with modeled δOR. Each trajectory was assembled into 10 clusters using the trajectory clustering protocol implemented in Desmond. The top five clusters with the most interacting members were further assessed using Prime MM-GBSA (Supplemental Tables S7 and S8). The top poses were further inspected and used for analyses and figures presented here.

## Figures and Tables

**Table 1:** Pharmacological characterization of compound **1**. All assays were run in three or more independent trials. ND = not detected.

Parameter	δOR	µOR	κOR
Affinity (pKi ± SEM)	5.94 ± 0.16	<5	<5
cAMP Potency (pIC_50_ ± SEM)	6.01 ± 0.09	<5	<5
β-ARR2 potency (pEC_50_)	<5	ND	ND
β-ARR1 potency (pEC_50_)	<5	-	-

**Table 2:** Potency (pIC_50_) and standard error (SEM) of analogs of compound **1** to inhibit cAMP signaling at δOR. The sigma catalog number for each compound is provided. All compounds were tested in three or more independent trials.

Compound	Sigma Catalog Number	pIC50 ± SEM
**1**	R995045	6.0 ± 0.1
**2**	R563412	4.9 ± 0.1
**3**	R723622	5.1 ± 0.2
**4**	R443638	4.9± 0.2
**5**	R442488	5.0 ± 0.1
**6**	R910759	4.9 ± 0.2
**7**	R994944	ND
**8**	R817031	5.0 ± 0.1
**9**	R563420	4.8 ± 0.1
**10**	R729426	5.1 ± 0.2
**11**	R731501	5.4 ± 0.1
**12**	R455865	5.1 ± 0.2
**13**	R728691	5.1 ± 0.1
**14**	R729639	5.0 ± 0.1
**15**	L262382	5.0 ± 0.4
**Leu^5^-enkephalin**	-	9.1 ± 0.1

## Data Availability

Not applicable.

## Supplementary Materials

The following are available, Figure S1: Binding sites within the δOR structure generated using SiteMap, Figure S2: *Cα* RMSD of δOR and compound **1** obtained from 3 independent MD simulations with varying trajectory time lengths and starting points, Figure S3: Receptor and ligand RMSD across several MD simulations, Figure S4: Summary of key δOR amino acid interactions with compound **1** and Leu-Enkephalin in the presence of compound **1**, Figure S5: Pharmacophore mapping analysis using the receptor-ligand complex. Figure S6: Comparison of the thermostabilized and simulated wild-type agonist-bound δOR structures. Table S1: Smiles of δOR agonists and antagonists used to validate the initial docking models, Table S2: Docking and glide scores for known δOR agonists and antagonists used to validate the initial docking model before structural optimization of the model δOR, Table S3: Docking and glide scores for known δOR agonists and antagonists used to validate the initial docking model after structural optimization, Table S4: Compound **1** docking scores using the SP scoring function. Top 10 poses were rescored XP scoring function, Table S5: Top 15 Leu-enkephalin poses docked into model δOR in the presence of compound **1**, Table S6: Rescoring of top 50 poses of Leu-enkephalin docked into model δOR using Prime MM-GBSA, Table S7: MM-GBSA scoring of top 5 clusters from a 300 ns MD simulation for Leu-enkephalin and compound **1**, Table S8: MM-GBSA scoring of top 5 clusters from a 300 ns MD simulation for compound **1**.

## Sample Availability

The compounds are available from commercial vendors e.g., Sigma.
