# Supplementary material for: Identification of a Novel Delta Opioid Receptor Agonist Chemotype with Potential Negative Allosteric Modulator Capabilities
Source: Molecules. 2021 Nov 29;26(23):7236. doi: 10.3390/molecules26237236 (PMC8659279; doi:10.3390/molecules26237236)
Supplement: Supplementary file 1 [file molecules-26-07236-s001.zip › molecules-1473070-supplementary.pdf]

Supplementary Figures and Tables

Title: Identification of a novel delta opioid receptor agonist chemotype, with potential negative allosteric modulator capabilities.

Authors: Yazan J. Meqbil[1,2], Hongyu Su[1], Robert J. Cassell[1,], Kendall L. Mores[1], Anna M Gutridge[1], Benjamin R. Cummins[3], Lan Chen[4], Richard M. van Rijn[1,4,5,‡]

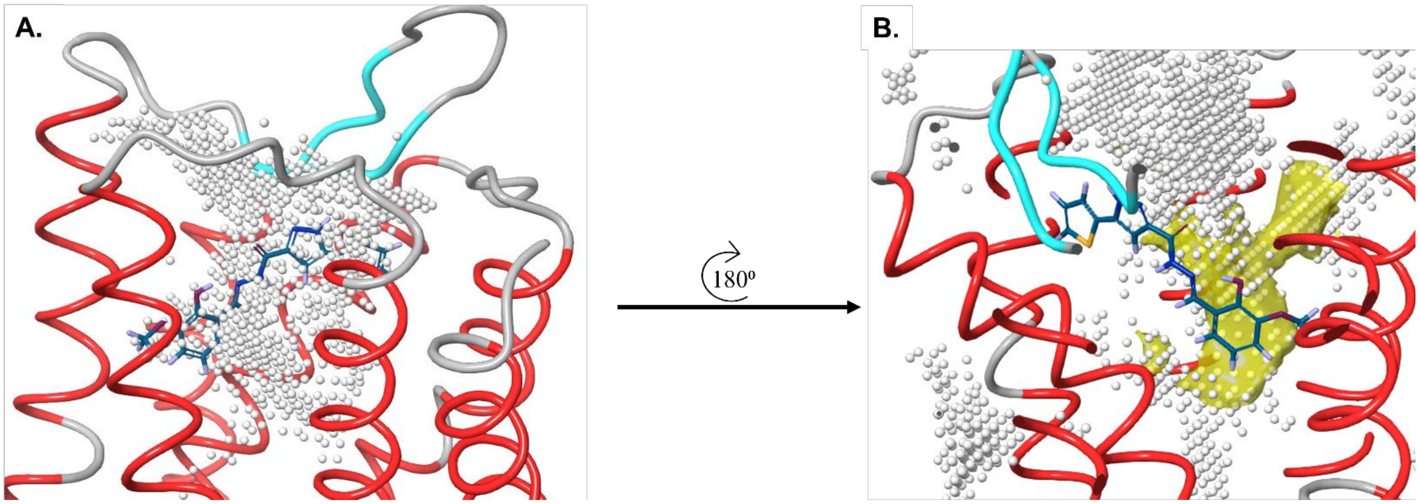

Supplementary Figure S1. Binding sites within the δOR structure generated using SiteMap. **A.** Compound 1 docked into the highest scoring binding site generated using SiteMap in the Schrödinger drug discovery suite (Schrödinger, Inc. NY) which confirmed similar interactions to our initial modeling. **B.** In the presence of compound 1, SiteMap predicted the Leu-enkephalin binding site (clustered in the yellow hydrophobic orthosteric site). This binding site was used in subsequent docking and MM-GBSA scoring prior to production MD simulations.

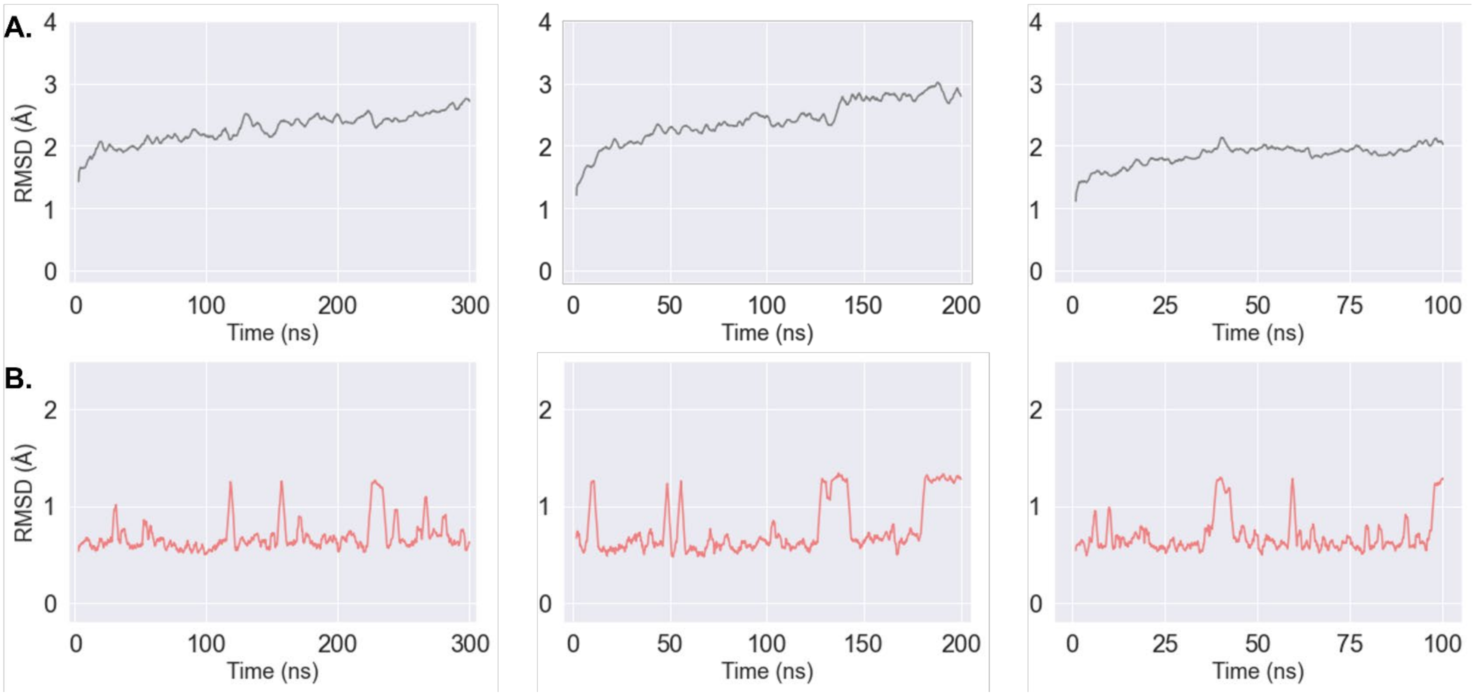

Supplementary Figure S2. *Cα* RMSD of δOR and compound 1 obtained from 3 independent MD simulations with varying trajectory time lengths and starting points (300ns, 200ns, 100ns, respectively). **A.** RMSD of δOR **B.** RMSD of compound 1. RMSD is represented as the rolling average every 3ns (11 frames).

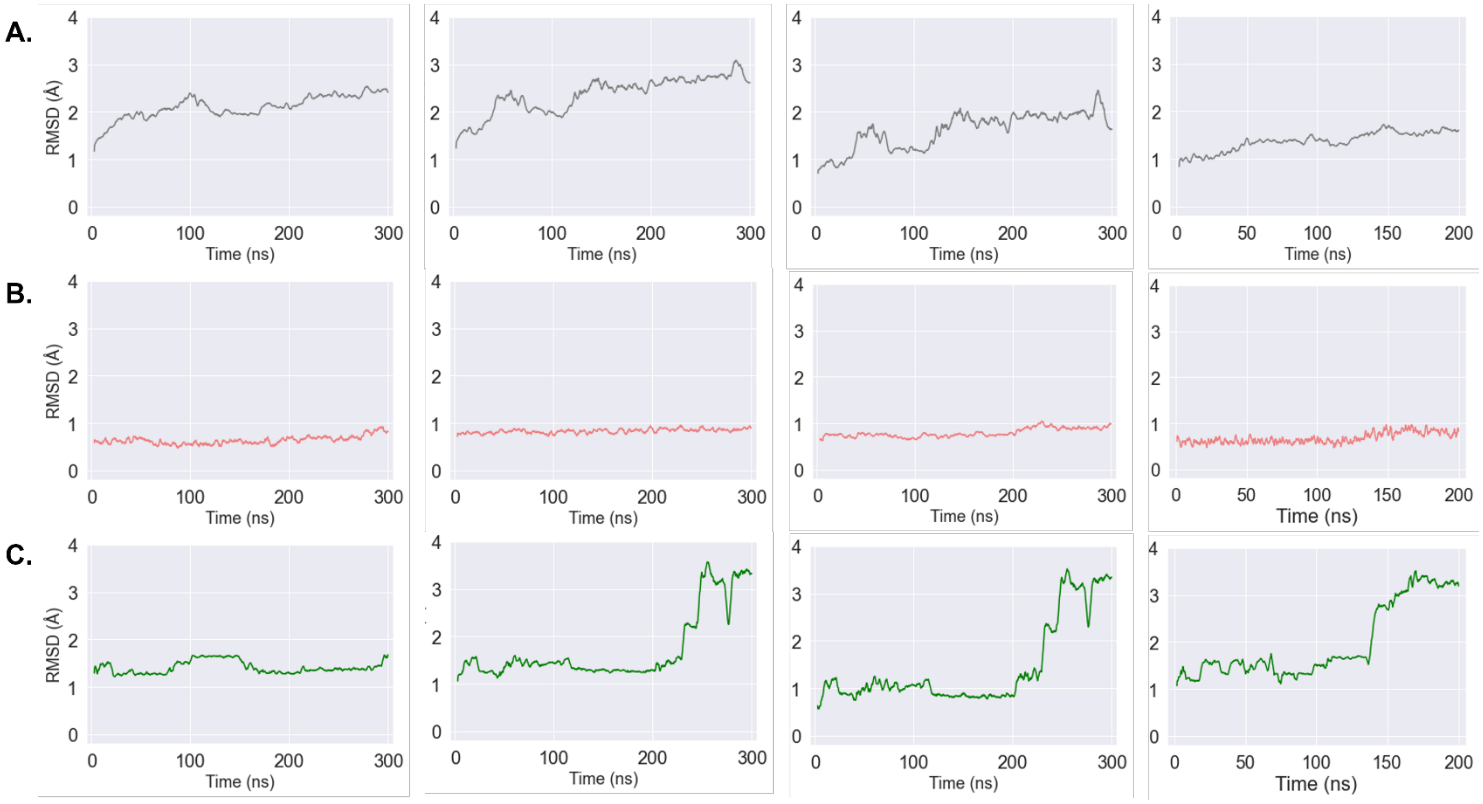

Supplementary Figure S3. Receptor and ligand RMSD across several MD simulations (300ns, 300ns, 300ns, 200ns, respectively). **A.** RMSD of δOR. **B.** RMSD of compound 1 in the presence of Leu-enkephalin. **C.** RMSD of Leu-enkephalin in the presence of compound 1. RMSD is represented as the rolling average every 3ns (11 frames).

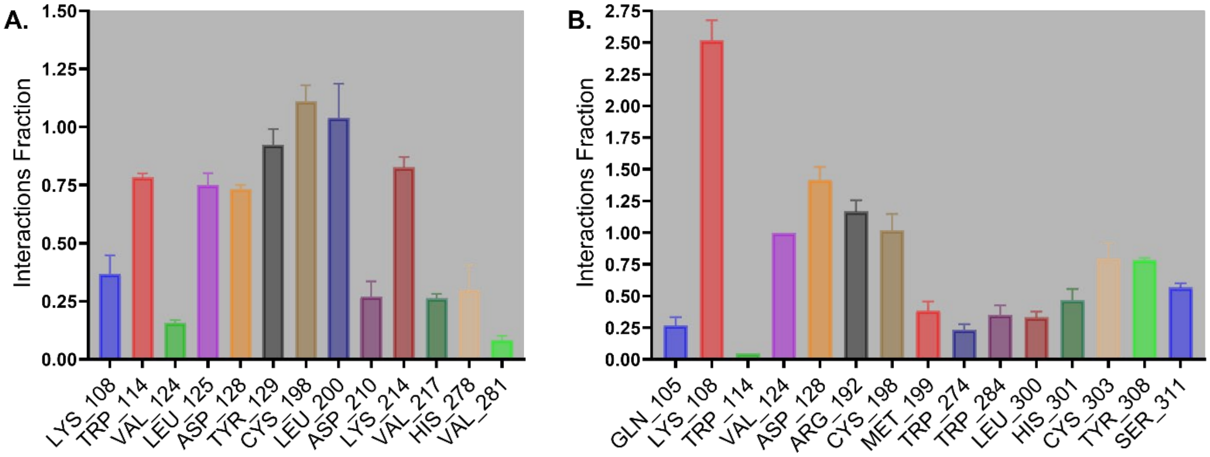

Supplementary Figure S4. Summary of key δOR amino acid interactions with compound 1 and Leu-Enkephalin in the presence of compound 1**.** Interactions fractions for **A.** compound 1 alone and **B.** Leu-enkephalin in the presence of compound 1. Fractions reported as the normalized mean ± SEM of at least 3 independent MD simulations.

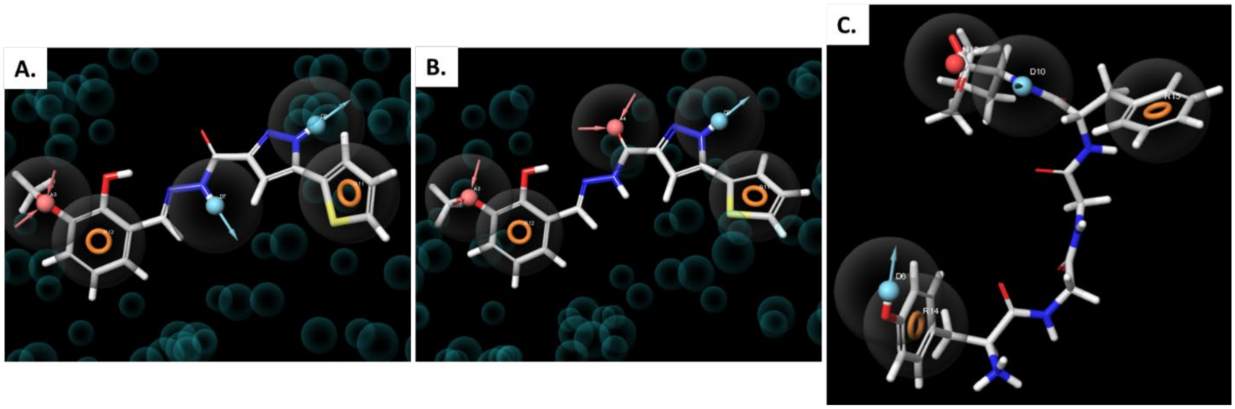

Supplementary Figure S5. Pharmacophore mapping analysis using the receptor-ligand complex. **A.** Compound 1 bound at the δOR exhibits two aromatic rings, two H-bond donors and one H-bond acceptor **B.** Compound 1 bound at the δOR in the presence of Leu-Enkephalin loses one H-donor but gains one H-acceptor **C.** Leu-Enkephalin pharmacophores in the presence of compound 1.

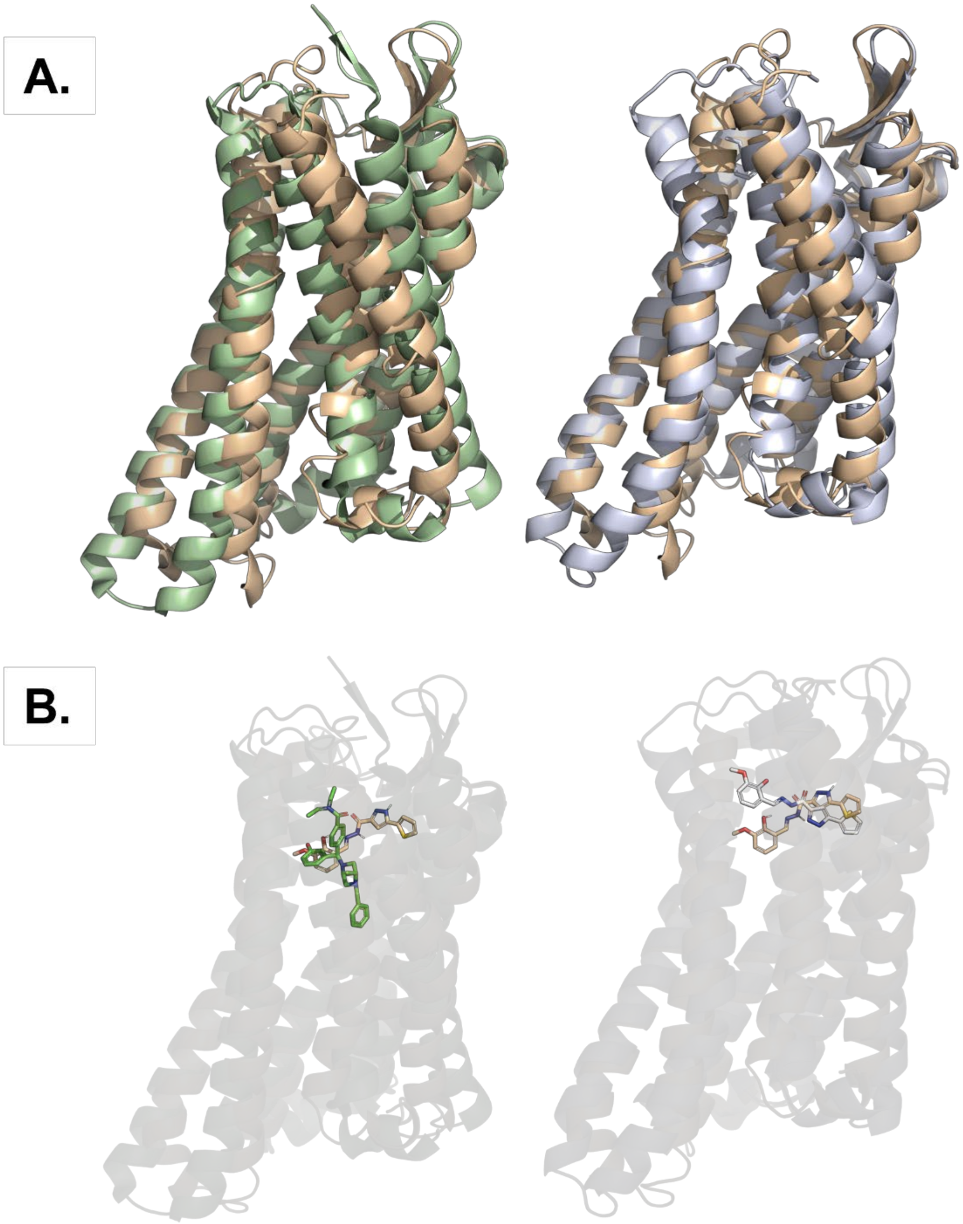

Supplementary Figure S6. Comparison of the thermostabilized and simulated wild-type agonist-bound δOR structures **A.** Model δORwt (receptor: wheat; compound 1: orange) superimposed on (left panel) the thermostabilized crystal structure of δOR (PDB: 6PT3, green) and (right panel) a representative structure obtained from a 300ns MD simulation using the thermostabilized crystal structure with compound 1 bound at δOR (blue white). **B.** Binding poses of DPI-287 (left panel, green) and compound 1 simulated at the thermostabilized structure (right panel, white).

Supplementary Table S1: Smiles of δOR agonists and antagonists used to validate the initial docking models.

Ligand	PUBCHEM_OPENEYE_CAN_SMILES
ADL5747	CCN(CC)C(=O)C1=CC(=C(C=C1)C2=CC3(CCNCC3)OC4=CC=CC=C42)O
ADL5859	CCN(CC)C(=O)C1=CC=C(C=C1)C2=CC3(CCNCC3)OC4=CC=CC(=C42)O.Cl
BU080828	CCC(C)(C)C(C)(C1CC23CCC1(C4C25CCN(C3CC6=C5C(=C(C=C6)O)O4)CC7CC7)OC)O
BW373U86	CCN(CC)C(=O)C1=CC=C(C=C1)C(C2=CC(=CC=C2)O)N3CC(N(CC3C)CC=C)C
DADLE	CC(C)CC(C(=O)O)NC(=O)C(CC1=CC=CC=C1)NC(=O)CNC(=O)C(C)NC(=O)C(CC2=CC=C(C=C2)O)N
DPDPE	CC1(C(C(=O)NCC(=O)NC(C(=O)NC(C(SS1)(C)C)C(=O)O)CC2=CC=CC=C2)NC(=O)C(CC3=CC=C(C=C3)O)N)C
DPI-287	CCN(CC)C(=O)C1=CC=C(C=C1)C(C2=CC(=CC=C2)O)N3CC(N(CC3C)CC4=CC=CC=C4)C
Leu-enk	CC(C)CC(C(=O)O)NC(=O)C(CC1=CC=CC=C1)NC(=O)CNC(=O)CNC(=O)C(CC2=CC=C(C=C2)O)N
Met-enk	CSCCC(C(=O)O)NC(=O)C(CC1=CC=CC=C1)NC(=O)CNC(=O)CNC(=O)C(CC2=CC=C(C=C2)O)N
SNC80	CCN(CC)C(=O)C1=CC=C(C=C1)C(C2=CC(=CC=C2)OC)N3CC(N(CC3C)CC=C)C
UFP-512	CC1=CC(=CC(=C1CC(C(=O)N2CC3=CC=CC=C3CC2C(=O)NC(CC(=O)O)C4=NC5=CC=CC=C5N4)N)C)O
Naloxone	C=CCN1CCC23C4C(=O)CCC2(C1CC5=C3C(=C(C=C5)O)O4)O
Naltrexone	C1CC1CN2CCC34C5C(=O)CCC3(C2CC6=C4C(=C(C=C6)O)O5)O
Naltrindole	C1CC1CN2CCC34C5C6=C(CC3(C2CC7=C4C(=C(C=C7)O)O5)O)C8=CC=CC=C8N6
Cyclazocine	CC1C2CC3=C(C1(CCN2CC4CC4)C)C=C(C=C3)O
Etorphine	CCCC(C)(C1CC23C=CC1(C4C25CCN(C3CC6=C5C(=C(C=C6)O)O4)C)OC)O
FR-140423	COC1=CC=C(C=C1)N2C(=CC(=N2)C(F)F)C3=CC=C(C=C3)S(=O)C
(-)-Bremazocine	CCC12CCN(C(C1(C)C)CC3=C2C=C(C=C3)O)CC4(CC4)O
DPI-3290	CC1CN(C(CN1C(C2=CC(=CC=C2)C(=O)N(C)C3=CC(=CC=C3)F)C4=CC(=CC=C4)O)C)CC=C
ARM-390	CCN(CC)C(=O)C1=CC=C(C=C1)C(=C2CCNCC2)C3=CC=CC=C3
SB219825	CCN1CCC2(CC3=C(CC2C1)C(=C(N3)C(=O)N(CC)CC)C)C4=CC(=CC=C4)O
DPI-221	CCN(CC)C(=O)C1=CC=C(C=C1)C(C2=CC=CC=C2)N3CC(N(CC3C)CC4=CC(=CC=C4)F)C
SB-235863	CC1=C(NC2=C1CC3C4CC5=C6C3(C2OC6=C(C=C5)OC)CCN4C)C(=O)OCC(C)C
BU-48	COC12C=CC3(C4C1C(CCC4)O)C5CC6=C7C3(C2OC7=C(C=C6)O)CCN5CC8CC8
AZD-2327	CCN(CC)C(=O)C1=CC=C(C=C1)C(C2=CC(=CC=C2)N)N3CCN(CC3)CC4=CC=C(C=C4)F
SB-227122	CC1=C(NC2=C1CC3C4CC5=C(C3(C2)CCN4C)C(=C(C=C5)OC)O)C(=O)N(C(C)C)C(C)C
SIOM	CN1CCC23C4C(=O)C5(CC6=CC=CC=C6C5)CC2(C1CC7=C3C(=C(C=C7)O)O4)O
JNJ20788560	CCN(CC)C(=O)C1=CC=C(C=C1)C(C2CC3CCC(C2)N3CCC4=CC=CC=C4)C5=CC=CC=C5
N-Desmethylclozapine	C1CN(CCN1)C2=NC3=C(C=CC(=C3)Cl)NC4=CC=CC=C42

Supplementary Table 2: Docking and glide scores for known δOR agonists and antagonists used to validate the initial docking model before structural optimization of the model δOR.

δOR Ligand	docking score	glide gscore	glide emodel
DPI287	-9.01	-9.21	-75.15
BW373U86	-7.59	-7.62	-51.21
KNT127	-7.53	-7.69	-59.59
Cyclazocine	-7.48	-7.49	-45.27
Etorphine	-7.40	-7.43	-60.35
TIPP	-7.38	-8.05	-65.82
JNJ20788560	-7.09	-7.09	-58.79
DPI3290	-6.99	-7.19	-60.91
BU-48	-6.29	-6.42	-53.02
SIOM	-6.29	-6.42	-53.02
SB219825	-5.97	-5.98	-51.77
SNC80	-5.69	-5.72	-56.78
BU080828	-5.67	-5.67	-52.78
ADL5859	-5.01	-5.01	-28.35
ARM390	-4.69	-4.72	-33.13
FR-140423	-4.67	-4.67	-40.22
AZD2327	-4.51	-4.55	-44.69

Supplementary Table 3: Docking and glide scores for known δOR agonists and antagonists used to validate the initial docking model after structural optimization.

δOR Ligand	docking score	glide gscore	glide emodel
**DPI-287**	-8.02	-8.22	-93.70
AZD-2327	-7.97	-8.17	-88.53
DPI-221	-7.82	-8.01	-84.44
JNJ20788560	-7.27	-7.27	-71.80
BW373U86	-7.14	-7.17	-66.79
DPI-3290	-6.77	-6.79	-63.36
ARM-390	-6.72	-6.72	-61.58
UFP-512	-6.66	-6.93	-84.93
SB219825	-6.57	-6.57	-63.71
SNC80	-6.44	-6.47	-66.40
CHEMBL3604280	-6.38	-6.43	-57.24
CHEMBL3604278	-6.33	-6.36	-60.30
Naltrindole	-6.26	-6.30	-55.24
(-)-Bremazocine	-6.19	-6.20	-48.10
N-Desmethylclozapine	-6.19	-6.20	-54.98
Naltrexone	-6.08	-6.12	-52.58
KNT-127	-6.02	-6.19	-53.66
Naloxone	-5.93	-6.05	-51.82
ADL5747	-5.91	-5.92	-56.87
CHEMBL3604282	-5.85	-5.87	-48.54
CHEMBL3604281	-5.82	-5.83	-57.83
FR-140423	-5.76	-5.76	-58.23
SIOM	-5.70	-5.82	-56.94
BU-48	-5.68	-5.70	-48.18
BU080828	-5.64	-5.64	-57.21
Etorphine	-5.57	-5.61	-57.54
Cyclazocine	-5.55	-5.55	-47.18
ADL5859	-5.49	-5.49	-52.62
SB-235863	-5.35	-5.38	-54.24
SB-227122	-5.28	-5.30	-52.05
CHEMBL3604279	-5.27	-5.31	-46.30
R995045	-5.02	-5.45	-56.27

Supplementary Table 4: Compound 1 docking scores using the SP scoring function. Top 10 poses were rescored XP scoring function.

Compound 1 poses	SP docking score	SP glide gscore	SP glide emodel	XP docking score	XP glide gscore	XP glide emodel
1	-5.28	-5.70	-60.44	-5.74	-6.16	-54.49
2	-5.01	-5.43	-52.16	-4.27	-4.70	-48.40
3	-2.81	-5.44	-53.24	-2.97	-5.60	-58.18
4	-2.79	-5.41	-56.87	-2.87	-5.50	-54.12

Supplementary Table 5: Top 15 Leu-enkephalin poses docked into model δOR in the presence of compound 1.

Leu-enkephalin poses	docking score	glide gscore	glide emodel
1	-9.66	-9.85	-107.84
2	-9.54	-9.74	-95.66
3	-8.83	-9.58	-105.74
4	-8.82	-9.02	-100.49
5	-8.75	-9.50	-109.13
6	-8.58	-8.78	-93.63
7	-8.24	-8.99	-102.71
8	-8.15	-8.90	-82.47
9	-7.84	-8.59	-90.48
10	-7.46	-7.66	-103.63
11	-7.46	-7.66	-91.03
12	-7.46	-7.65	-84.03
13	-7.44	-7.63	-88.46
14	-7.42	-7.61	-85.64
15	-7.39	-7.59	-88.17

Supplementary Table 6: Rescoring of top 50 poses of Leu-enkephalin docked into model δOR using Prime MM-GBSA.

Leu-enkephalin poses	MMGBSA dG Bind
1	-84.79
2	-84.22
3	-82.32
4	-81.29
5	-79.49
6	-74.44
7	-73.72
8	-72.85
9	-71.66
10	-71.03
11	-70.20
12	-68.57
13	-66.06
14	-64.65
15	-64.50

Supplementary Table 7: MM-GBSA scoring of top 5 clusters from a 300ns MD simulation for Leu-enkephalin (LE) and compound 1 (cmpd 1).

Cluster-Ligand	MMGBSA dG Bind
18 Members_LE	-69.18
18 Members_cmpd 1	-63.74
14 Members_LE	-62.06
14 Members_cmpd 1	-61.95
11 Members_LE	-70.16
11 Members_cmpd 1	-63.08
10 Members_LE	-70.59
10 Members_cmpd 1	-61.84
9 Members_LE	-65.39
9 Members_cmpd 1	-58.21

Supplementary Table 8: MM-GBSA scoring of top 5 clusters from a 300ns MD simulation for compound 1 (cmpd 1).

Cluster-Ligand	MMGBSA dG Bind
25 Members_ligand	-60.88
10 Members_ligand	-59.7
10 Members_ligand	-57.77
8 Members_ligand	-56.9
8 Members_ligand	-58.66
